# Altered structural covariance of the cortex and hippocampal formation in patients with lung cancer after chemotherapy

**DOI:** 10.1016/j.heliyon.2024.e40284

**Published:** 2024-11-13

**Authors:** Renyuan Liu, Ping Rong, Yiming Ma, Pin Lv, Ningyu Dong, Wenqian Chen, Fan Yang, Qiuyue Zhao, Shangwen Yang, Ming Li, Xiaoyan Xin, Jiu Chen, Xin Zhang, Xiaowei Han, Bing Zhang

**Affiliations:** a Department of Radiology, Nanjing Drum Tower Hospital, Affiliated Hospital of Medical School, Nanjing University, China; b Institute of Medical Imaging and Artificial Intelligence, Nanjing University, China; c Medical Imaging Center, The Affiliated Drum Tower Hospital, Medical School of Nanjing University, China; d Department of Radiology, The Quzhou Affiliated Hospital of Wenzhou Medical University, Quzhou People's Hospital, Quzhou, China

**Keywords:** Brain, Chemotherapy, Gray matter volume, Lung cancer, Magnetic resonance imaging, Structural covariant network

## Abstract

**Objective:**

In this retrospective study, we aimed to investigate changes in brain morphology and structural topological networks in patients with lung cancer (LC) with or without chemotherapy.

**Methods:**

We retrospectively recruited 191 participants for this cross-sectional study, including 113 patients with LC without chemotherapy (Ch-), 38 patients with LC with chemotherapy (Ch+), and 40 healthy controls (HC) matched for age, sex, and education. The gray matter volume (GMV) and cortical properties were compared among the three groups. We constructed the structural covariant network (SCN) based on cortical thickness, volumes of subcortical structures, and volumes of hippocampal subfields and the amygdala in all participants. The global and nodal topological properties of SCN were compared among groups. In addition, 23 patients with LC (8 Ch+ and 15 Ch-) who received two identical brain magnetic resonance scans were enrolled in the follow-up study. The paired *t*-test was used to compare group differences in brain morphology and topological properties in the structural network.

**Results:**

The GMV of the bilateral caudate and thalamus were smaller in the Ch- and Ch + groups compared to the HC group using threshold-free cluster enhancement and permutation (*P* < 0.05, 5000 times permutations) for multiple comparison correction. The cortical SCN analysis suggested multiple enhanced nodal properties in several brain areas in Ch+ and Ch-compared to HC, mainly in the temporal gyrus, using permutations test and false discovery rate (FDR) (*P* < 0.05) corrections. Moreover, an increased sigma was found in the Ch + compared with HC (*P* = 0.0238). The reduced nodal degree (*P* = 0.0002) and betweenness (*P* = 0.0008) in the right amygdala of Ch + compared to HC were detected by subcortical SCN analysis. Furthermore, reduced gamma (*P* = 0.0342) and sigma (*P* < 0.0001) were found in Ch-compared with HC in the SCN analysis of subfields of the amygdala-hippocampal complex. In the follow-up study, reduced nodal degree (*P* < 0.0001) was found in the right anterior amygdala, and reduced clustering coefficient and local efficiency were found in patients with LC after the permutation test.

**Conclusions:**

Our study showed GMV defects and structural topological property abnormalities related to LC and chemotherapy. Such morphological changes associated with LC and chemotherapy could be used as imaging markers for clinical assessments and pathological indicators.

## 1 Introduction

Lung cancer (LC) is a carcinoma with the highest morbidity and mortality worldwide, which severely reduces the quality of life and the life expectancy of patients [1,2]. In particular, the number of patients with LC in China has been increasing over the years, and according to statistics, approximately one million people may die from LC by 2025 [3].

Platinum-based chemotherapy has been regarded as the standard treatment for lung malignancies, and it can effectively prolong the life of patients [[4], [5], [6]]. However, several studies have focused on the neurotoxic mechanisms related to chemotherapy and have reported subsequent brain damage [[7], [8], [9]]. Apple et al. [10] found that cancer survivors receiving chemotherapy had decreased gray matter (GM) volume in the bilateral hippocampi. Moreover, patients experienced more apparent GM atrophy in the frontal lobe area after chemotherapy [11,12]. Recently, researchers have applied neuroimaging techniques, especially structural magnetic resonance imaging (MRI), and found that patients with LC have aberrant brain morphology compared to healthy controls (HC) [13]. Marta [14] used voxel-based morphometry (VBM) analysis and reported lower GM density in the insula and bilateral parahippocampal gyrus in patients with LC who underwent chemotherapy. In addition, patients with LC who received chemotherapy presented with atrophic cortical thickness and volume in the orbitofrontal cortex, middle temporal gyrus, and precuneus [15].

Structural covariant network (SCN) analysis is another approach generally used to assess the integrity of brain structural networks [16]. In recent years, the research on brain functional connectivity network (FCN) impairments associated with LC and chemotherapy has increased [[17], [18], [19]]. Although evidence suggests a high spatial overlap between the SCN and FCN [20,21], few studies have investigated SCN damage patterns in patients with LC receiving chemotherapy.

In this retrospective study, we used multiple structural neuroimaging analysis methods to adequately detect the pattern of GM defects in patients with LC, including GM volume, cortical thickness, and structural topological properties. We hypothesized that patients with LC, whether with or without platinum-based chemotherapy, exhibit altered morphology and structural network inconsistencies in multiple brain regions compared with HC.

## 2 Methods

### 2.1 Participants

**Fig. 1:**
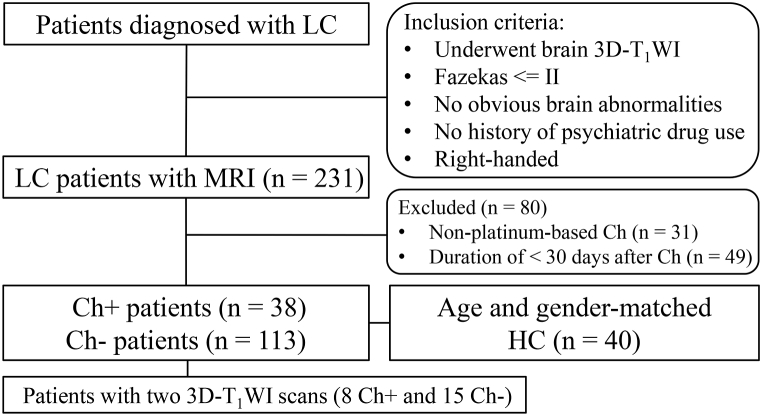
Flow chart of the study. LC: lung cancer. Ch: chemotherapy. Ch+: patients with platinum-based chemotherapy. Ch-: patients without platinum-based chemotherapy. HC: healthy control.

All participants were retrospectively enrolled at our hospital between January 2019 and June 2021. The inclusion criteria were as follows: 1) patients diagnosed with lung cancer based on pathological results; 2) patients with (Ch+) or without (Ch-) platinum-based chemotherapy; 3) patients who underwent at least one 3D-T_1_WI; 4) patients with no other brain or psychiatric diseases and no history of psychotropic drug use; and 5) right-handed patients. The exclusion criteria were as follows: 1) patients with brain metastasis, stroke, arterial aneurysm, cerebral hemorrhage, obvious brain atrophy, and Fazekas score > II; 2) patients with a duration of <30 days after chemotherapy; and 3) patients who underwent non-platinum chemotherapy (Fig. 1).

In the longitudinal study, we identified 23 patients with LC (8 Ch+ and 15 Ch-) who received two 3D-T_1_WI scans. None of the patients had brain metastasis, stroke, obvious brain atrophy, or other imaging appearances that met the exclusion criteria in both scans; therefore, they were all included in the follow-up study.

The inclusion criteria for HC were as follows: 1) participants with one 3D-T_1_WI; 2) individuals with no history of systemic diseases or neurological symptoms and signs; 3) individuals with no history of psychiatric diseases and psychotropic drug use; 4) individuals with no appearance of stroke, arterial aneurysm, cerebral hemorrhage, obvious brain atrophy; 5) individuals with Fazekas score ≤ II; and 6) right-handed individuals.

Images were evaluated by two radiologists with >5 years of working experience, and the Ch+, Ch −, and HC groups were matched for age, sex, and years of education. This study was approved by the Medical Ethics Committee of the Nanjing Drum Tower Hospital. The data are anonymous, and the requirement for informed consent was therefore waived.

### 2.2 Image acquisition

Images were acquired using a 3.0T MR scanner (Ingenia CX; Philips) with a 32-channel head coil. The 3D-T_1_WI images were acquired using a three-dimensional fast-spoiled gradient-echo sequence, with the following parameters: repetition time/echo time: 6.6 ms/3.0 ms; flip angle, 8°; field of view, 250 mm × 250 mm × 180 mm; matrix, 250 × 250; slice thickness, 1 mm; gap, 0 mm; averages, 1.

### 2.3 Voxel-based morphometry

VBM and group-wise comparisons were performed using fmrib software library (FSL) (v6.0; https://fsl.fmrib.ox.ac.uk/fsl/fslwiki/). Briefly, brain extractions and segmentations were performed on all T1 images using the “fslvbm_1_bet” command; then, participants with decent brain extraction results and mild morphological changes were selected for generating the study-specific GM template. We selected 11 participants from each group to form the GM template using the “fslvbm_2_template” command. Non-linear registrations of all GM images were performed by the command “fslvbm_3_proc” to form the 4D GM image for statistical analysis. Group-wise comparisons, using age as a covariate, were performed by the command “randomise,” with threshold-free cluster enhancement (TFCE) method for cluster detections. Differences among the three groups were analyzed by ANOVA, followed by post-hoc analysis. A permutation test was used for multiple comparison corrections, with a significance level set at *P* < 0.05, with 5000 permutations. In the follow-up study, a paired *t*-test was used to detect differences between the groups, with the same significance level mentioned above.

### 2.4 Vertex-based analysis

FreeSurfer v7.2.0 (http://surfer.nmr.mgh.harvard.edu/) was used for image processing and vertex-based analysis. Briefly, the FreeSurfer workflow includes three parts: preprocessing steps, such as motion correction, intensity normalization, and skull strip; subcortical segmentation and tessellation of the pial surface and GM white matter boundary; volumetric quantification of cortical and subcortical segmentation. The FreeSurfer pipeline was run with the command line “recon-all” with default options, which resampled the images into 1 mm^3^ for further processing. Group-wise analysis was conducted with the command line “mri_glmfit,” with the option “-sim” for Monte Carlo Simulation for multiple comparison corrections. Differences among the three groups were analyzed by ANOVA, followed by post-hoc analysis. In multiple comparisons corrections, the vertex-wise and cluster-wise significance levels were set to *P* < 0.001 and *P* < 0.05, respectively. For surface-based analysis, participants’ cortical maps were generated to the FreeSurfer standard template and smoothed with a 10-mm full-width half-maximum Gaussian kernel by the command line “recon-all -qcahe”. Then, a general linear model analysis was performed on the template surface with age and intracranial volumes as covariates. In the follow-up study, a paired *t*-test was used to detect differences between the groups, with the same significance level mentioned above.

### 2.5 Structural covariance network analysis

The brain connectivity toolbox (https://sites.google.com/site/bctnet/) was used to construct the SCN and calculate network properties. The SCN was constructed based on Pearson's correlations between all possible pairs of structural regions of interest (ROIs), and only positive correlations were included in network construction. ROIs were defined as SCN nodes, whereas Pearson's correlations between ROI pairs were defined as SCN edges. Before SCN construction, linear regression was performed at every ROI to remove the effects of age and intracranial volumes, and the resulting residuals were used as raw ROI values for SCN analysis. A sparsity thresholding sequence (threshold range, 0.05–0.40, with increments of 0.01) was applied to normalize the number of edges and calculate the area under the curve (AUC) for each network property. The range of the sparsity thresholding sequence was determined by ensuring that >90 % of the SCN matrices resemble the small-worldness (sigma >1.1).

Structural covariance properties were calculated based on a group-specific structural covariance matrix, thus yielding a single value of each network property from each group. The topological properties of SCN can be described and quantified as the characteristic path length (Lp), clustering coefficient (Cp), global efficiency, and nodal topological properties, such as nodal degree centrality, betweenness, and nodal efficiency. A thousand random networks were generated to calculate the random Cp and random Lp, which were used to calculate the normalized Cp (gamma) and normalized Lp (lambda) that were eventually used to calculate sigma, a parameter commonly used for quantifying the small-world topology.

A permutation test, with the significance level and number of permutations set to *P* < 0.05 and 5000 times, respectively, was used to determine the significance of observed group differences in structural covariance properties. The participants were shuffled between the two groups multiple times by the permutation test to generate a distribution of randomized group differences. Thus, yielding the probability of getting data as extreme as the observed data when the null hypothesis is true. Additional false discovery rate (FDR) corrections (*q* < 0.05) were used for nodal property comparisons. In the follow-up study, permutations were only conducted in each patient pair, with the same permutation times and significance levels mentioned above.

### 2.6 ROIs selection for SCN

In this study, we performed SCN analysis separately for three volumetric properties: cortical thickness, volumes of subcortical structures, and volumes of hippocampal subfields and nuclei of the amygdala. For cortical SCN, ROIs were defined by the Desikan-Killiany Atlas, which contains 68 cortical ROIs from both hemispheres. The cortical thickness of each ROI was generated using the command line “aparcstats2table.” To validate the bias caused by different cortical parcellations, we also applied the Destrieux Atlas for SCN construction, which would perform structural covariance analysis on cortical thickness based on 148 ROIs from bilateral hemispheres.

For subcortical SCN, to simplify the FreeSurfer segmentation results, we only included the bilateral thalamus, caudate, putamen, pallidum, hippocampus, amygdala, and the posterior, mid-posterior, central, mid-anterior, and anterior parts of the cingulate cortex, thus generating a 17 × 17 Pearson's correlation matrix for SCN construction.

For structural covariance analysis of hippocampal subfields and nuclei of the amygdala, ROIs were defined by the probabilistic atlas FreeSurfer used for subfield segmentation, which included 28 subfields from the hippocampus and amygdala, thus formed a 56 × 56 Pearson's correlation matrix for SCN construction. Command line “segmentha_t1.sh” was used to segment hippocampal subfields and amygdala nuclei. Only T1 images were used for segmentation of the subfields. Then, the “asegstats2table” command was used to generate the volumes of each structural ROI.

### 2.7 Statistical analysis

Demographic and clinical characteristics statistics were analyzed with analysis of variance using SPSS (version 21.0), with a significance criterion of *P* < 0.05 (2-tailed). Inter-group differences in sex were analyzed using Chi-square tests.

## 3 Results

### 3.1 Participant characteristics

In the cross-sectional study among the three groups, 191 participants were retrospectively enrolled, including 113 patients with LC without chemotherapy, 38 patients with LC with chemotherapy, and 40 well-matched HC. In addition, 23 patients with LC were included in the follow-up study, with an interval of 0.33 ± 0.17 years. Details of the demographics and clinical data of the participants are listed in Table 1.

**Table 1:** Demographic and clinical characteristics of the study population.

		Cross-sectional			P value	Follow-up
		HC	Ch+	Ch-		
Gender (male/female)		24/16	31/7	78/35	0.114	15/8
Age (mean ± SD)		59.7 ± 9.38	62.2 ± 8.6	62.2 ± 9.92	0.213	61.0 ± 9.39
Education (years, mean ± SD)		9.0 ± 4.8	8.6 ± 4.6	7.3 ± 4.5	0.080	8.31 ± 3.74
Histological types (cases/proportion)	Squamous cell carcinoma	–	16 (42.1)	31 (27.4)	–	8 (34.7)
	Adenocarcinoma	–	13 (34.2)	73 (64.6)	–	12 (52.1)
	Other	–	10 (23.7)	9 (8)	–	3 (13.2)
Chemotherapy cycle (cycles, mean ± SD)		–	4.7 ± 3.8	–	–	7.4 ± 9.9
Duration after chemotherapy (days, mean ± SD)		–	345.2 ± 462.7	–	–	330.5 ± 399.6
Interval of chemotherapy (years, mean ± SD)		–	–	–	–	0.41 ± 0.30

### 3.2 Subcortical atrophy detected by voxel-based morphometry

**Fig. 2:**
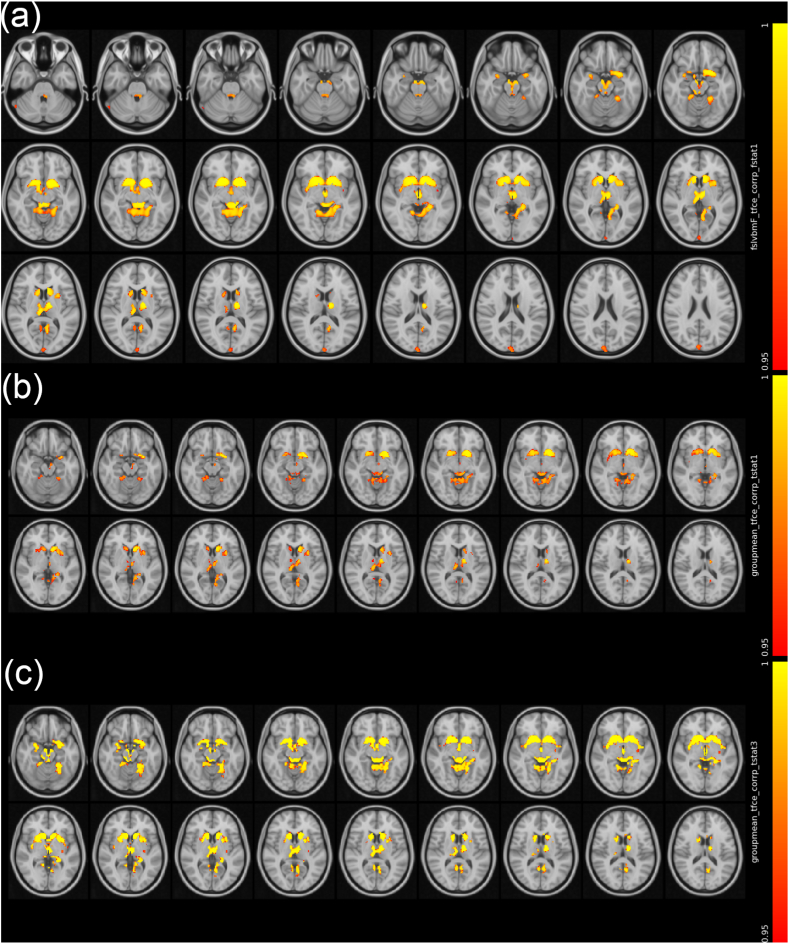
Group differences in VBM detected by ANOVA (a), with *P* < 0.05 and TFCE corrections. Significant atrophy of the bilateral caudate and thalamus was found in both (b) Ch- and (c) Ch+, compared to HC. Color bar: corrected P value (1-P for ease of display). (For interpretation of the references to colour in this figure legend, the reader is referred to the Web version of this article.)

Significant and varied differences in subcortical structural morphometry were observed among the groups using VBM. After TFCE corrections, significant atrophy (*P* < 0.05) of the bilateral caudate and thalamus was found in both Ch+ and Ch-, in comparison with HC (Fig. 2A–C); no subcortical volumetric differences between Ch+ and Ch-remained significant after TFCE corrections. No obvious cortical differences were found among the groups with VBM.

### 3.3 Cortical properties remained intact under vertex-based analysis

Based on vertex-wise group comparisons, no significant differences in cortical thickness were found between the groups after Monte Carlo Simulation corrections (vertex *P* < 0.001, cluster *P* < 0.05). Accordingly, no differences in curvature, area, or other cortical structural properties were observed.

### 3.4 Inconsistent results of cortical SCN

**Fig. 3:**
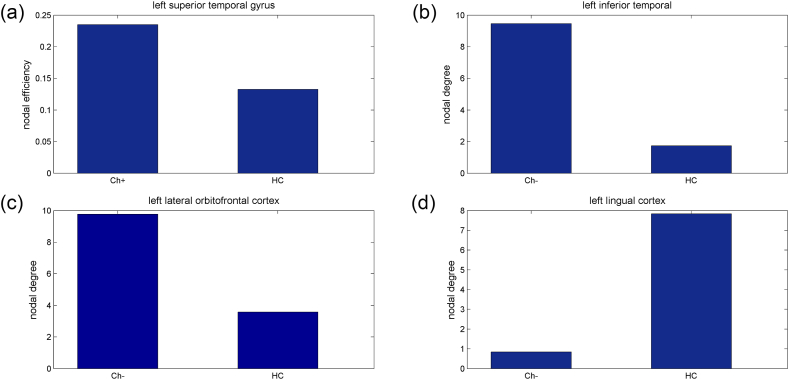
Cortical SCN based on the Desikan-Killiany Atlas indicated inconsistent topological changes among the groups. In comparison with the HC group, (a) enhanced nodal efficiency (*P* < 0.001, FDR <0.05) was found in the left superior temporal gyrus in the Ch + group, while increased (b–c) and a reduced (d) nodal degree (all three *P* values = 0.002, FDR <0.05) were observed in the Ch-group.

Cortical SCN analysis based on the Desikan-Killiany Atlas showed enhanced nodal efficiency in the left superior temporal gyrus in the Ch + group compared to that in the HC group (Fig. 3A). Meanwhile, an enhanced nodal degree was found in the left inferior temporal and lateral orbitofrontal cortex of the Ch-group (Fig. 3B–C) and a reduced nodal degree was found in the left lingual cortex (Fig. 3D), in comparison to that in the HC group. All differences remained significant after the permutation test and FDR correction. The global topological properties revealed no significant differences among the groups.

**Fig. 4:**
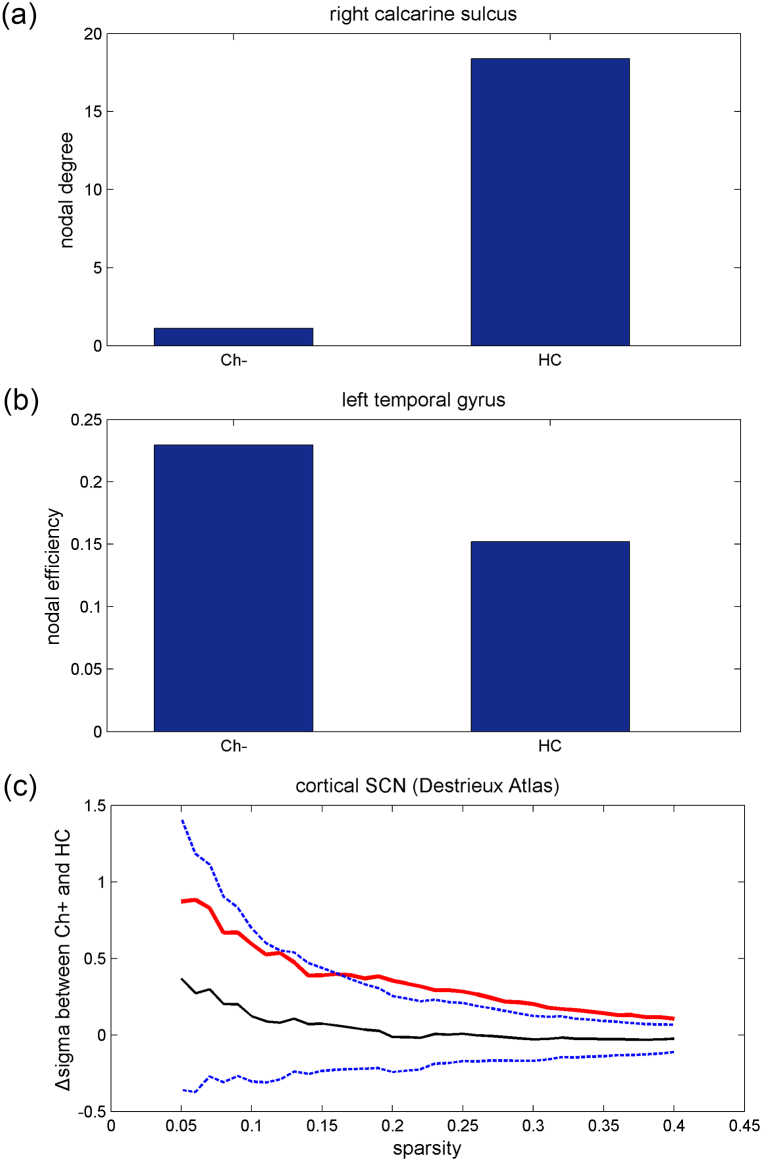
Cortical SCN with the application of the Destrieux Atlas: (c) significantly enhanced sigma was found in the Ch + group (*P* = 0.0238, FDR <0.05) compared with that in the HC group. Red line: the differences of sigma between Ch+ and HC under sequential sparsity threshold; black line: the differences of sigma between Ch+ and HC from permutation tests; blue line: 95 % confidence interval. Meanwhile, reduced nodal degree (a) and enhanced nodal efficiency (b) were found in the Ch-group in comparison with the HC group (both *P* < 0.001, FDR <0.05). (For interpretation of the references to colour in this figure legend, the reader is referred to the Web version of this article.)

However, with the application of the Destrieux Atlas, no significant differences in nodal topological properties were found between the HC and Ch + groups. However, reduced nodal degree was found in the right calcarine sulcus of the Ch-group (Fig. 4A) compared to the HC group, while enhanced nodal efficiency was found in the left temporal gyrus (Fig. 4B). For global topological properties, significantly enhanced sigma was found in the Ch + group (*P* = 0.0238) compared with that in the HC group (Fig. 4C). No differences in global SCN properties were observed between the Ch and HC groups.

### 3.5 Reduced nodal properties of right amygdala detected by subcortical SCN

**Fig. 5:**
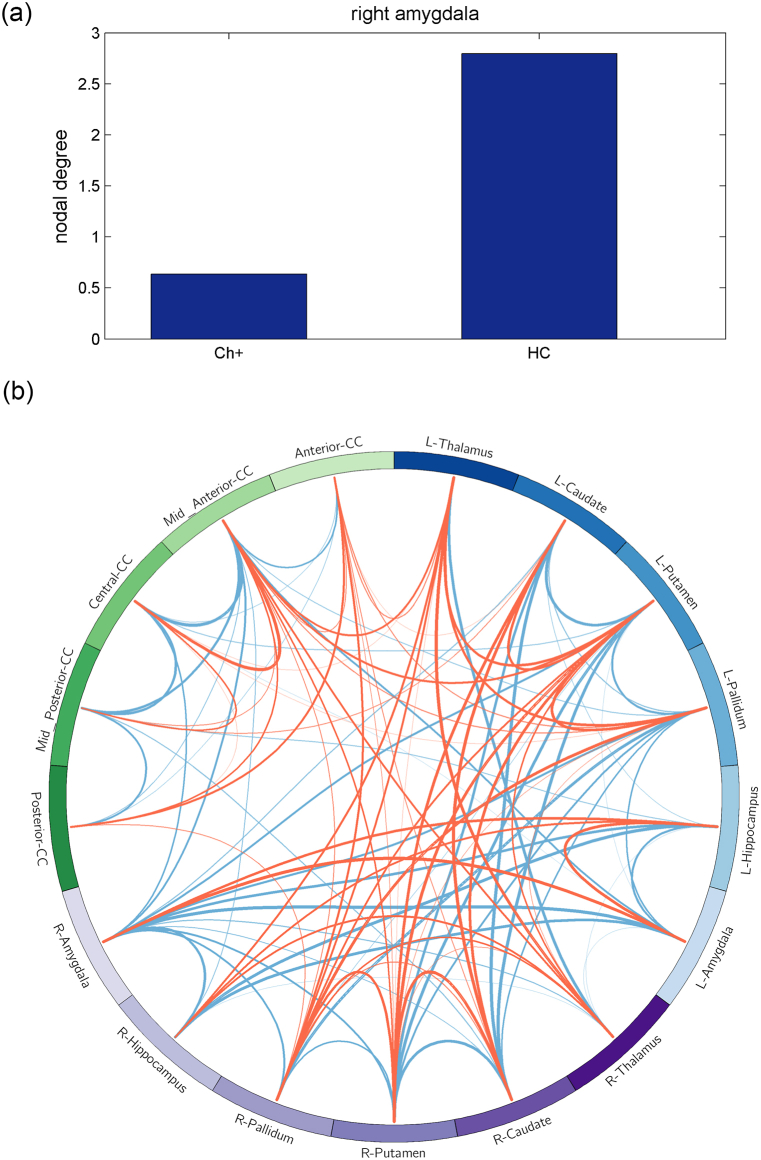
Subcortical SCN revealed reduced nodal degree (*P* = 0.0002) and betweenness (*P* = 0.0008) of the right amygdala of the Ch + group (both FDR <0.05). (b) Red line: group average nodal connections, obtained by one-sample *t*-test (uncorrected *P* < 0.001), of the Ch + group; blue line: group average nodal connections of the HC group. (For interpretation of the references to colour in this figure legend, the reader is referred to the Web version of this article.)

**Fig. 6:**
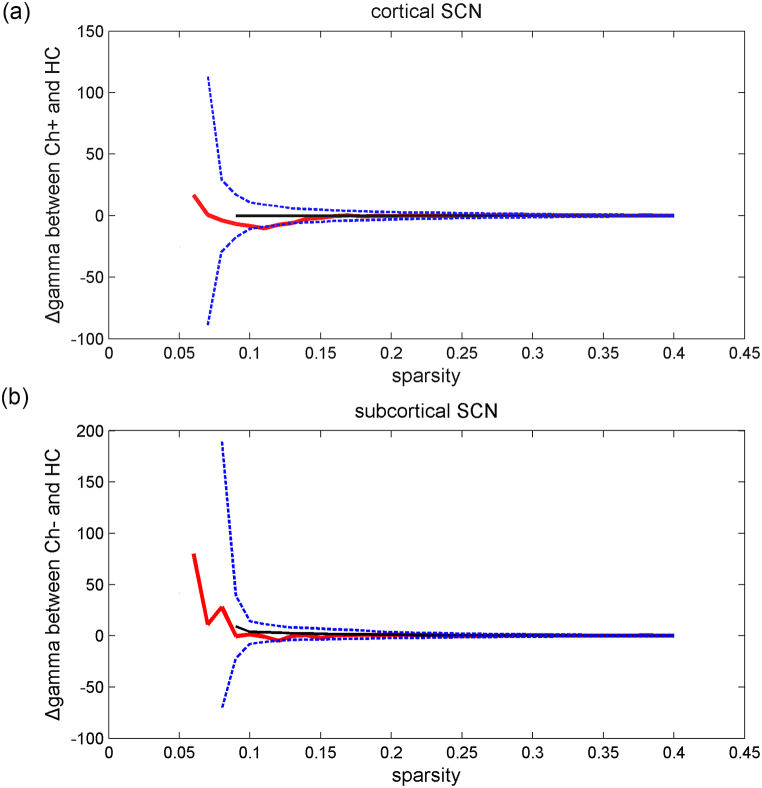
Subcortical SCN indicated a trend of reduced gamma was detected in both Ch+ (a) and Ch- (b) groups (both *P* < 0.0001) in comparison to that in the HC group. Red line: the differences of sigma between Ch+ and HC under sequential sparsity threshold; black line: the differences of sigma between Ch+ and HC from permutation tests; blue line: 95 % confidence interval. (For interpretation of the references to colour in this figure legend, the reader is referred to the Web version of this article.)

Subcortical SCN analysis revealed reduced nodal degree (*P* = 0.0002) and nodal betweenness (*P* = 0.0008) in the right amygdala of the Ch + group compared to that in the HC group (Fig. 5A and B). No significant differences in nodal properties were observed between the Ch- and HC groups. For global SCN characteristics, a trend of reduced gamma was detected in both Ch+ and Ch-groups (both *P* < 0.0001) in comparison to that in the HC group (Fig. 6A and B), which resulted in reduced sigma in the Ch+ and Ch-groups.

### 3.6 Reduced SCN properties of amygdala-hippocampal complex

**Fig. 7:**
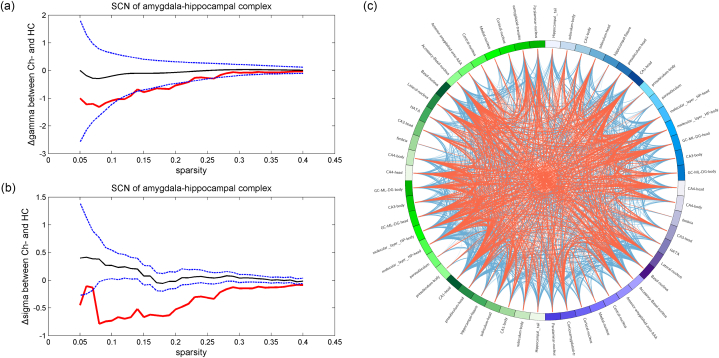
SCN of amygdala-hippocampal complex detected reduced gamma (*P* = 0.0342) and sigma (*P* < 0.0001) of the Ch-group compared to that in the HC group (both FDR <0.05). (a–b) Red line: the differences of gamma and sigma between Ch- and HC under sequential sparsity threshold; black line: the differences of gamma and sigma between Ch- and HC from permutation tests; blue line: 95 % confidence interval. (c) Sparse nodal connections of the amygdala-hippocampal complex were also observed in the Ch-group compared to that in the HC group. Red line: group average nodal connections, obtained by one-sample *t*-test (uncorrected *P* < 0.001), of the Ch-group; blue line: group average nodal connections of the HC group. (For interpretation of the references to colour in this figure legend, the reader is referred to the Web version of this article.)

No significant volumetric differences in the hippocampal subfields and amygdala nuclei were found among the groups after FDR correction. However, based on SCN analysis of subfields of the amygdala-hippocampal complex, reduced gamma (*P* = 0.0342) and sigma (*P* < 0.0001) were found in the Ch-group compared to that in the HC group (Fig. 7A–C). Both *P* values remained statistically significant after the permutation test. No differences in SCN nodal properties were observed between the Ch- and HC groups. For comparisons between the Ch+ and HC groups, no significant group-wise differences were found in either global or nodal SCN properties.

### 3.7 Reduced nodal SCN properties of the right amygdala area observed in the follow-up study

**Fig. 8:**
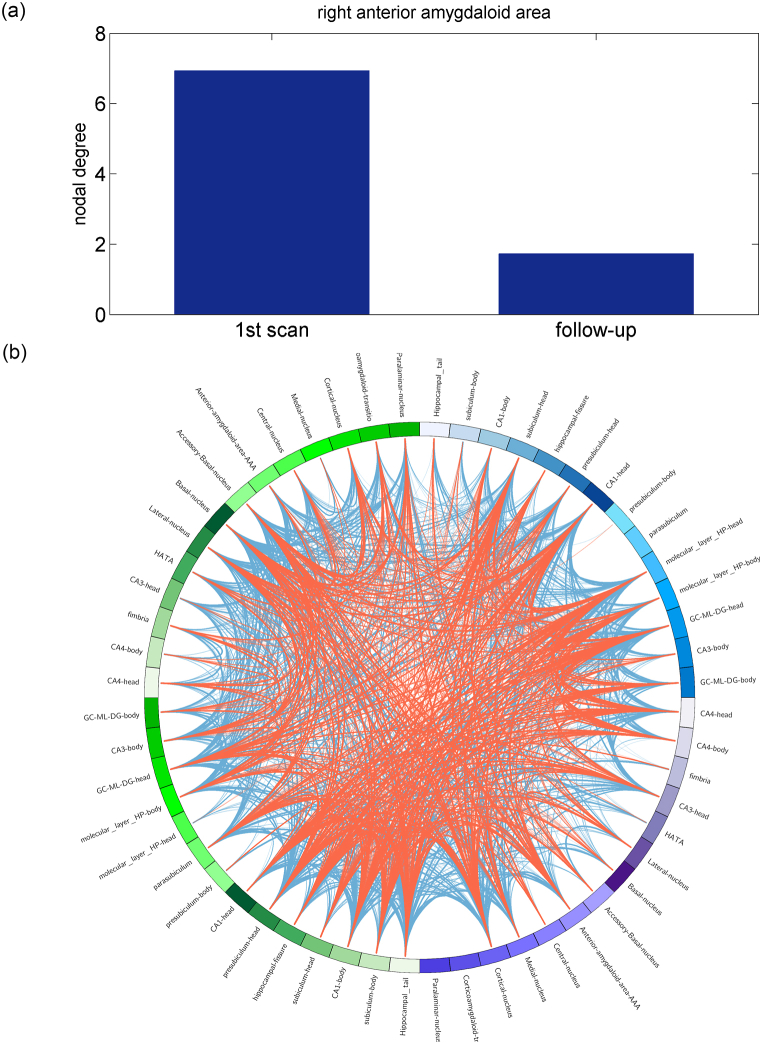
Follow-up SCN of amygdala-hippocampal complex detected a significant reduction (*P* < 0.001, FDR <0.05) in the nodal degree of the right anterior amygdala area (a). Sparse regional connections revealed by reduced clustering coefficient (*P* = 0.0208) and local efficiency (*P* = 0.0224) were also observed in the follow-up scan compared with that in the initial scan (b). Red line: group average nodal connections, obtained by one-sample *t*-test (uncorrected *P* < 0.001), of the follow-up scan; blue line: group average nodal connections of the initial scan. (For interpretation of the references to colour in this figure legend, the reader is referred to the Web version of this article.)

For 23 patients (8 Ch+ and 15 Ch-) who underwent two 3D-T_1_WI scans, no significant differences in cortical and subcortical volumetric changes were found between the two scans by VBM and vertex-based analysis. Additionally, no significant differences in the cortical and subcortical SCN were found between the two scans. However, in SCN analysis of the hippocampal-amygdala complex, a significant decrease in the nodal degree of the right anterior amygdala area (Fig. 8A) was found in the follow-up scan (*P* < 0.0001). Furthermore, reduced clustering coefficient (*P* = 0.0208) and local efficiency (*P* = 0.0224), both of which remained significant after permutation tests, were also found in the follow-up scan (Fig. 8B), indicating interrupted morphometric changes in cancer biology and platinum-based chemotherapy.

## 4 Discussion

In addition to symptoms of the respiratory system, abnormal neuroimaging findings in patients with LC, especially those receiving chemotherapy, have recently become of great concern [22,23]. In the current study, we found significant atrophy in the bilateral caudate and thalamus in patients with LC, whether with or without chemotherapy, using the VBM approach. Cortical SCN analysis revealed nodal property abnormalities in multiple brain areas due to LC and chemotherapy. Meanwhile, subcortical SCN analysis detected reduced nodal features in the right amygdala in the chemotherapy group. Decreased global topological properties of the subfields of the amygdala-hippocampal complex were also detected by the SCN analysis in the non-chemotherapy group compared to HCs. In the longitudinal study including 23 patients with LC, whether with or without platinum-based chemotherapy, results showed decreased nodal degree in the right anterior amygdala area, and reduced clustering coefficient and local efficiency in the follow-up scan compared to the first scan.

The results of GM volume comparison among the three groups suggested a significant decrease in the volume of the caudate and thalamus in chemotherapy and non-chemotherapy groups, which is consistent with a previous study [7]. The caudate and thalamus are crucial structures that are vulnerable to LC invasion [24,25]. Zhang [26] has explored higher cerebral blood flow (CBF) and lower CBF connectivity in the caudate in patients with LC after chemotherapy. A recent resting-state functional MRI study has reported a change in neural activity coherence in the caudate associated with LC and chemotherapy [27]. Moreover, imbalanced functional connections of the thalamus have been observed in patients with LC [28]. Therefore, combining our findings with previous studies, the caudate and thalamus may be highly sensitive to LC and are vulnerable to damage during the early stage of cancer. Notably, the volume of caudate and thalamus decreased more significantly in the chemotherapy group, indicating that chemotherapy could further accelerate volume atrophy in several brain regions [29,30].

The amygdala and hippocampus are crucial subcortical structures that participate in modulating distinct emotions and memories [[31], [32], [33]] and are regarded as important hubs of the whole brain network [34,35]. In the SCN analysis of the subfields of the amygdala-hippocampus complex, we showed reduced gamma and sigma levels in patients with LC without chemotherapy. Gamma and sigma are the parameters set to define the small-world nature of the human brain [36,37]. The reduced small-world index represents disrupted brain network structures and inefficient information transmission between the subfields of the amygdala and hippocampus [38]. Several studies have suggested that a disordered functional topological network of the amygdala and hippocampus is related to LC [17,28]. Considering the direct relationship between the brain's functional and structural connections [39], the current findings further demonstrate the vulnerabilities of the amygdala and hippocampus to LC. In the subcortical SCN analysis, we found that the nodal degree and betweenness of the right amygdala were lower in the Ch + group than in the HC group. However, there were no significant differences between the Ch- and HC groups. Furthermore, in the follow-up study, we also found lower nodal degree in the right anterior amygdala area in patients with LC, whether with or without chemotherapy, compared to HC. The altered global or local topological properties of the amygdala indicate fewer interactions between the amygdala and other brain areas, which may influence information processing and delivery [40]. The special impact of chemotherapy was consistent with the common theory of chemotherapy neurotoxicity [41,42]. The biomechanisms of chemotherapy-related neurotoxicity are diverse. The platinum compounds could interact with DNA and lead to neuronal apoptosis [43,44]. In contrast, the chemotherapeutic agents cause neuronal degeneration by activating multiple pro-inflammatory cytokines and inducing oxidative stress [45].

In the cortical SCN analysis, several enhanced nodal features were found in the Ch- and Ch + groups, mainly in the temporal gyrus. Meanwhile, an increased sigma was found in Ch+. Therefore, there may be a compensatory mechanism that could compensate the impaired structural organization in other brain regions associated with LC and chemotherapy to some extent. Further studies are required to confirm this hypothesis.

This study has several limitations. First, due to the absence of cognitive indicators, we were unable to determine individual behavioral changes caused by brain structural disorders. Chemotherapy-related cognitive decline is often overlooked in daily clinical practices. Therefore, during the therapy sections, no cognitive assessments were acquired from the LC patients of this study. In the future, we plan to assess various cognitive functions of participants and identify correlations between cognition and brain structural changes. Secondly, the retrospective nature of this study resulted in a limited sample size and inaccurate criteria for each group. For example, including individuals with Fazekas ≤ II as HC may introduce mild white matter abnormalities within the HC group, thus compromising the conclusions of chemotherapy-related morphological changes observed in this study. However, in order to comply with the Fazekas status of LC patients and to maintain sufficient participants in the HC group, a wider range of Fazekas results was ultimately applied to the HC inclusion criteria. The inaccuracy of including both Ch+ and Ch-patients in the longitudinal analysis may also introduce a mixed effect of LC-related and chemotherapy-related gray matter changes in the observation of this study, thus the cause for abnormal morphological and SCN changes has yet to be identified. Overcoming these limitations can be achieved through a larger sample size or a well-designed cohort study, which is crucial for verifying our findings. Third, unlike diffusion-based or functional-based MR analysis, SCN analysis does not provide direct connections between brain regions. No fiber tracts nor functional correlations could be obtained between the abnormal brain regions detected by the SCN analysis. Therefore, brain regions with volumetric abnormalities detected by SCN analysis could only be considered as potential imaging markers for clinical assessments and indications of underlying brain networks or circuits.

This study combined the analysis approaches of GM volume and SCN to explore abnormal neuroimaging in patients with LC. We revealed alterations in brain morphology and structural connections in patients with LC with or without chemotherapy. Additionally, we found atrophic brain parenchyma and disordered topological associations among brain regions. Our results provide increased knowledge about the neural mechanisms underlying LC- and chemotherapy-related brain impairment. The morphological neuroimaging findings involving GM atrophy and structural covariance abnormalities may be considered as potential neuroimaging markers for LC assessments and indicators for chemotherapy protection.

## CRediT authorship contribution statement

**Renyuan Liu:** Writing – review & editing, Writing – original draft, Methodology, Formal analysis. **Ping Rong:** Writing – original draft, Data curation. **Yiming Ma:** Investigation, Data curation. **Pin Lv:** Investigation. **Ningyu Dong:** Investigation, Data curation. **Wenqian Chen:** Investigation, Data curation. **Fan Yang:** Data curation. **Qiuyue Zhao:** Data curation. **Shangwen Yang:** Supervision, Project administration. **Ming Li:** Supervision, Project administration. **Xiaoyan Xin:** Supervision, Resources, Project administration. **Jiu Chen:** Supervision, Resources, Conceptualization. **Xin Zhang:** Supervision, Resources, Conceptualization. **Xiaowei Han:** Writing – review & editing, Writing – original draft, Supervision, Project administration, Conceptualization. **Bing Zhang:** Writing – review & editing, Supervision, Project administration, Conceptualization.

## Ethics statement

All procedures performed in this study involving human participants were in accordance with the ethical standards of the Medical Ethics Committee of the Nanjing Drum Tower Hospital (approval number: 2020-379-01). Written informed consent was obtained from each participant.

## Data availability statement

Data associated with this study has not been deposited into a publicly available repository. However, all data will be made available on request.

## Declaration of AI and AI-assisted technologies in the writing process

No AI and AI-assisted technologies were applied during the preparation and writing process of this work.

## Funding statement

This work was supported by the National Science and Technology Innovation 2030 -- Major program of "Brain Science and Brain-Like Research" (2022ZD0211800); the 10.13039/501100001809National Natural Science Foundation of China (82102015, 82171908, 82271965, 81971596, 82001793); the 10.13039/501100012226Fundamental Research Funds for the Central Universities, 10.13039/501100004193Nanjing University (2020–021414380462); the Key Scientific Research Project of Jiangsu Health Committee (K2019025); Industry and Information Technology Department of Nanjing (SE179-2021); Educational Research Project of 10.13039/501100007289Nanjing Medical University (2019ZC036); Key Project supported by Medical Science and technology development Foundation, Nanjing Department of Health (ZKX21031), and fundings for Clinical Trials from the Affiliated Drum Tower Hospital, Medical School of Nanjing University (2021-LCYJ-PY-36, 2022-LCYJ-MS-25). Project of Modern Hospital Management and Development Institute, Nanjing University, and Aid project of Nanjing Drum Tower Hospital Health, Education & Research Foundation (NDYG2021005). The funders had no role in the study design, data collection and analysis, decision to publish, or preparation of the manuscript.

## Declaration of competing interest

The authors declare that they have no known competing financial interests or personal relationships that could have appeared to influence the work reported in this paper.
